# Long-Term Outcomes of 1989 Immediate Implant-Based Breast Reconstructions: An Analysis of Risk Factors for Failure and Revision Surgery

**DOI:** 10.1097/PRS.0000000000011744

**Published:** 2024-09-24

**Authors:** Merel M. L. Kooijman, Annelotte C. M. van Bommel, Frederieke H. van Duijnhoven, Astrid N. Scholten, Carolien H. Smorenburg, Leonie A. E. Woerdeman, Corstiaan C. Breugem

**Affiliations:** Amsterdam, the Netherlands; From the Departments of 1 Plastic and Reconstructive Surgery; 2 Surgical Oncology; 3 Radiotherapy; 4 Oncology, Netherlands Cancer Institute–Antoni van Leeuwenhoek Hospital; 5 Department of Plastic Surgery, Amsterdam University Medical Center.

## Abstract

**Background:**

Nipple- or skin-sparing mastectomy and immediate implant-based breast reconstruction (IBR) is potentially associated with long-term unfavorable outcomes, such as revision surgery and reconstruction failure. This large patient cohort study aimed to provide long-term data on the incidence of these outcomes and to identify predictive risk factors.

**Methods:**

Between 2012 and 2019, 1989 mastectomies with IBR were performed in 1512 women in the authors’ institute. A direct-to-implant method was used in 93% and a 2-staged method with tissue expander in 7%. Logistic regression analysis was used to identify patient- and treatment-related risk factors associated with revision surgery or reconstructive failure.

**Results:**

The mean follow-up was 62.2 months. IBR failed in 6.7% of all breasts; thus, a breast was present in 93.3%. Age older than 44 years yielded a 2.6-fold, and radiotherapy, a 1.7-fold increased risk for reconstruction failure. Revision surgery was performed in 60% of all breasts. The mean number of revisions of all IBRs was 1.2 (range, 0 to 8; SD, 1.37). Factors associated with significantly higher rates of revision surgery were age older than 44 years (OR, 1.23), smoking (OR, 1.53), specimen weight greater than 492 g (OR, 1.39), implant volume greater than 422 g (OR, 1.95), and radiotherapy (OR, 1.51). Nipple preservation was protective for both outcomes (OR, 0.71 and 0.42, respectively). Direct-to-implant procedures did not require any surgical revision in 43% of these patients.

**Conclusions:**

Despite the necessity of revision surgery in the majority of IBRs, nearly half of the breasts did not require any revision surgery, and long-term reconstruction failure rates are extremely low. Therefore, IBR should be offered to all eligible women undergoing mastectomy, while understanding the risks.

**CLINICAL QUESTION/LEVEL OF EVIDENCE:**

Risk, III.

Breast cancer treatment guidelines worldwide recommend that breast reconstruction should be offered to all women undergoing mastectomy, allowing shared decision-making.^1–4^ Immediate rather than delayed breast reconstruction allows these women to better cope with their mastectomy with improvement of quality of life in terms of self-image, self-esteem, and sexuality.^5–8^ Combined with nipple- or skin-sparing mastectomy, immediate reconstruction allows the preservation of native mammary skin envelope and the inframammary fold.

Immediate reconstruction can be performed using an implant or autologous tissue. Implant-based breast reconstruction offers technical and logistic advantages as compared to autologous reconstruction.^5,9^ An immediate implant-based breast reconstruction (IBR) is completed either with the direct-to-implant (DTI) approach or with the temporary use of a tissue expander first. In the DTI approach, a permanent prosthesis is inserted immediately, with the advantage to achieve a final result in 1 procedure.^10^ Using a tissue expander for a 2-staged approach gives the opportunity for additional adjustments during the second procedure.^10,11^

Either method may require revision surgery and both are associated with complications. Short-term complications include infection, skin necrosis, hematoma, and implant loss^12–18^; pain, distortion, capsular contracture, implant rupture, or unfavorable cosmetic outcomes are reported long-term unfavorable outcomes.^10,18–21^ Various risk factors for unfavorable outcomes associated with IBR have been reported,^10,14,15,18–21^ but data on long-term outcomes regarding revision surgery, reconstruction failure, and oncologic follow-up are scarce.

The main objectives of this study were to assess the long-term outcomes of IBR in patients undergoing mastectomy for breast cancer or prophylactic surgery and to assess risk factors for reconstruction failure or revision surgery.

## PATIENTS AND METHODS

### Patient Selection

All women treated in our institute with a skin- and nipple-sparing therapeutic or prophylactic mastectomy between January of 2012 and January of 2019 were prospectively included. Patients were referred to the plastic surgeon by the oncologic breast surgeon to discuss IBR if mastectomy was indicated. Patients with all 3 previously defined exclusion criteria,^14^ body mass index (BMI) greater than 25 kg/m², smoking, and expected breast volume of 500 g or greater or cup size E or larger were ineligible for IBR because of an unacceptably high risk of loss. Patients receiving a different implant brand than Allergan or who had previous chest wall radiation were excluded from this study, except for patients who received mantle field radiation therapy for Hodgkin lymphoma, because there is no increased risk of adverse surgical outcomes, as described in recent studies.^22,23^

### Surgical Procedure

Since 2000, a standardized technique of skin-sparing mastectomy and IBR has been routinely performed by 5 dedicated oncologic breast surgeons and 6 dedicated plastic surgeons in our institute.^14^ Since January of 2014, the nipple-areola complex is routinely preserved during mastectomy except when there is oncologic involvement or an expected risk of malpositioning, such as in grade 3 ptosis.^24–27^ Mastectomy is performed through an infra-areolar incision with lateral extension by the oncologic breast surgeon. Consecutively, the plastic surgeon releases the pectoralis major muscle from the origin up to the fourth intercostal space to enable subpectoral implant insertion. This lift is done to allow a more natural projection of the reconstructed breast and to prevent cranial displacement.^28^

In our institute, we favor a DTI approach in which a prosthesis is inserted. During the time examined in this study, we used Natrelle 410, a textured, high-cohesive gel–filled prosthesis (Allergan). If a larger breast was desired than would fit in the available skin envelope or if the pectoralis major muscle would not reach below the new nipple position, a saline solution–filled tissue expander (Natrelle 133; Allergan) was used. As of 2019, implants by Allergan are no longer used.

After implant insertion, the released pectoralis major origin is stabilized by suturing it to the subcutaneous tissue of the caudal skin flap. No biologic dermal matrix or synthetic mesh is used. Antibiotic prophylaxis for 24 hours and wound drainage for 5 to 14 days is applied in all patients.

### Outcome Measures

**Fig. 1.**
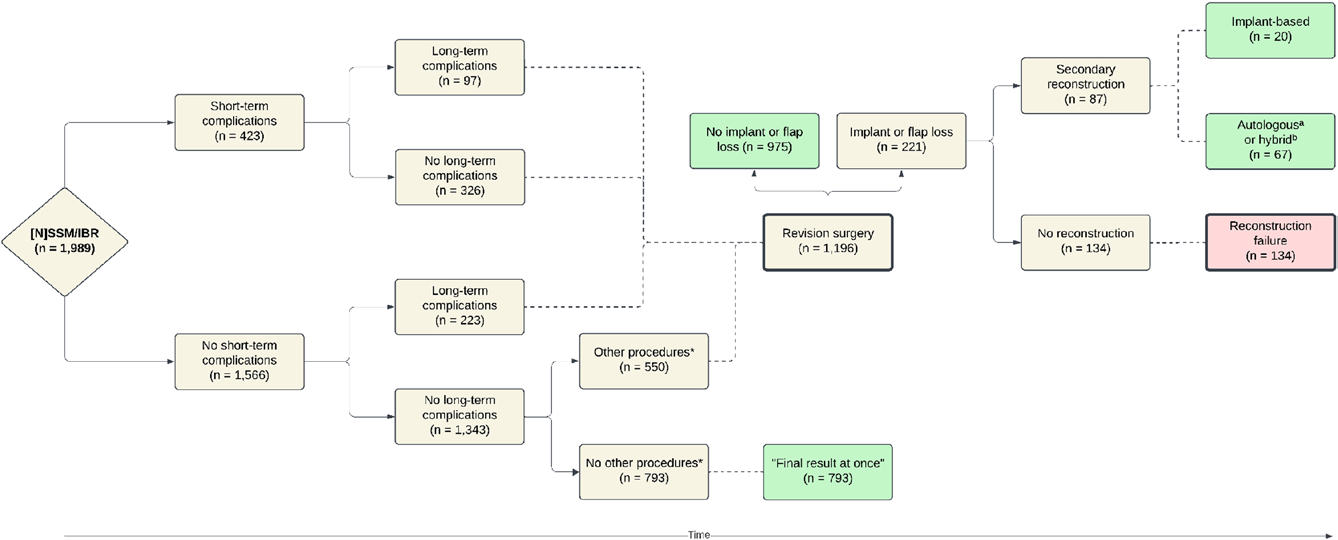
Distribution of 1989 immediate breast reconstructions following mastectomy over the various interventional paths. Note that 793 breasts (40%) had their reconstruction finished at once (“final result at once”), 1855 cases (93%) had a breast at end of follow-up (indicated in *green*), and 134 (6.7%) showed absence of a breast at end of follow-up (indicated in *red*). *Other procedures = any elective reintervention for aesthetic corrections of the breast, elective autologous or hybrid reconstructions, or revisions for oncologic purposes. ^a^With autologous reconstruction, solely autologous tissue (eg, deep inferior epigastric perforator flap, transverse rectus abdominis flap, or latissimus dorsi flap) is used. ^b^A hybrid reconstruction denotes combining autologous tissue transfer (latissimus dorsi flap or thoracodorsal flap) with the use of an implant. The *bolded* blocks represent the outcome measures. The *continuous lines* represent the division of groups into distinct outcomes over time. The *dashed lines* indicate the aggregation of groups into a specific cluster. *[N]SSM/IBR*, nipple- or skin-sparing mastectomy and immediate implant-based breast reconstruction.

Revision surgery and reconstruction failure were assessed as outcome measures (Fig. 1). Revision surgeries requiring general anesthesia were categorized into 3 groups. The first group consisted of short- and long-term complications that required surgical reintervention. Short-term complications were registered as reinterventions in the first 16 weeks postoperatively due to infection, hematoma, seroma, or necrosis. Long-term complications included complications after 16 weeks and reinterventions for implant malposition or distortion, mainly due to capsular contracture. The second group consisted of elective reinterventions for preferential purposes or aesthetic reasons. These elective reinterventions comprised lipofilling, liposuction, scar revision, scheduled replacement of a temporary tissue expander by a permanent prosthesis, and secondary elective autologous or hybrid reconstructions. Secondary reconstructions were performed after previous implant loss, after capsular contracture problems, or based on patient preference. In the third group, revisions for oncologic purposes were assessed (ie, interventions to eliminate a tumor-positive nipple or any residual or recurrent local tumor). In the Netherlands, medically indicated surgical treatments following IBR are reimbursed by insurance after authorized approval. The outcome “reconstruction failure” was defined as permanent absence of a breast at end of study.

Of all baseline characteristics (Table 1), 7 patient-related and 13 treatment-related characteristics were assessed as potential risk factors for revision surgery and reconstruction failure. (**See Table, Supplemental Digital Content 1**, which shows data on the prevalence and percentages of revision surgery [1196 breasts] and reconstruction failures [947 breasts] in a cohort of 1989 breasts that underwent IBR after mastectomy. The data are stratified by the presence or absence of potential risk factors, including, among others, age, BMI, smoking status, implant volume, and adjuvant radiation therapy, http://links.lww.com/PRS/H417.)

**Table 1.** Baseline Characteristics of 1989 Nipple- or Skin-Sparing Mastectomies Combined with Immediate Implant-Based Breast Reconstruction

Characteristics	Value
Patient-related, no. (%)	
Age, mean ± SD (range), yrs	43.7 ± 11.00 (19–76)
<35 years	518 (26)
35–44 years	599 (30)
45–54 years	604 (30)
>55 years	268 (14)
BMI, mean ± SD (range), kg/m^2^	23.6 ± 3.70 (15.0–43.4)
>25 kg/m^2^	558 (28)
Tobacco use	197 (9.9)
Comorbidity^a^	375 (19)
Specimen weight, mean ± SD (range), g	491.5 ± 264.83 (77–2100)
Prophylactic mastectomy	751 (38)
Therapeutic mastectomy	1238 (62)
<Stage IIA	739 (37)
≥Stage IIIA	208 (10)
Nonstaged^b^	291 (15)
Preoperative treatment-related, no. (%)	
Previous breast surgery^c^	284 (14)
Breast-conserving surgery^d^ (no radiotherapy)	173 (8.7)
Neoadjuvant systemic therapy	697 (35)
Chemotherapy	639 (32)
HER2 therapy	61 (3.1)
Hormonal therapy	72 (3.6)
Mantle field radiation treatment	33 (1.7)
Procedure-related, no. (%)	
Bilateral mastectomy (in women)	477 (24)
Nipple-areola complex preservation	1048 (53)
Direct-to-implant	1843 (93)
Implant volume, mean ± SD (range), g	421.5 ± 133.70 (50–775)
Nonstaff oncologic surgeon (resident or fellow)	326 (16)
Nonstaff plastic surgeon (resident or fellow)	283 (14)
Procedure time, mean ± SD (range), min	142.9 ± 43.76 (50–633)
Axillary lymph node procedure^e^	1030 (52)
Separate axillary incision	503 (49)
Postoperative treatment–related, no. (%)	
Adjuvant treatment	
Systemic therapy^f^	825 (42)
Chemotherapy	278 (14)
Anti-HER2 therapy	212 (11)
Hormonal therapy	653 (33)
Radiotherapy	384 (19)

a General health factors, such as diabetes; cardiovascular, pulmonary, thyroid, or hematologic disorders; concurrent oncologic disease; or active inflammatory diseases.

b Ductal carcinoma in situ (*n* = 280), phyllodes tumor (*n* = 2), a solitary metastasis of lung carcinoma (*n* = 1), melanoma (*n* = 1), (angio)sarcoma (*n* = 2).

c Breast surgery in general, such as augmentation or reduction, including breast-conserving surgery.

d Breast-conserving surgery, such as wide local excision.

e Axillary lymph node dissection, sentinel node or marking axillary lymph nodes with radioactive iodine procedure.^46^

f Some women underwent more than 1 modus of adjuvant systemic therapy.

To provide a comprehensive overview of all long-term outcomes of our study population, we also assessed the oncologic follow-up.

### Statistical Analysis

All analyses were calculated per breast, as the outcome measures are related to the specific breast rather than the patient in case of bilateral surgery. The 2-tailed Pearson chi-square or Fisher exact test was used to compare the prevalence of unfavorable outcomes for all dichotomous variables that can act as potential risk factors. All continuous variables were reduced to dichotomous variables, as the data were normally distributed. Univariable logistic regression analysis was performed for all factors potentially influencing the separate outcomes. *P* values less than 0.05 were accepted as indicative of statistical significance. Only the risk factors that demonstrated significant *P* values in the univariable analysis and were compliant with the Holm-Bonferroni correction method, to account for multiple comparisons, were subjected to multivariable logistic regression analysis.^29^ We evaluated the goodness of fit of the multivariable logistic regression model using the Hosmer-Lemeshow test, which resulted in a *P* value of 0.54 for revision surgery and 0.39 for reconstruction failure, indicating a good fit to the data. All statistical analyses were performed using SPSS (version 29.0; SPSS, Inc.).

## RESULTS

### Patient Selection

Of all 2416 breasts (1920 women) requiring mastectomy between January of 2012 and January of 2019, a total of 1999 (1519 women) underwent IBR. This results in an 83% immediate reconstruction rate in our institute (79% of all women). Ten breasts (7 women) were excluded; therefore, 1989 IBRs in 1512 women were included for further analysis. Mean age was 43.7 years, mean BMI was 23.6 kg/m^2^, and 10% were smokers. Therapeutic mastectomy was performed in 62% of all breasts (*n* = 1238); the remaining received a prophylactic mastectomy. Nipple-sparing mastectomy was performed in 53% and DTI reconstruction in 93% of all breasts. Mean follow-up was 62.2 months (range, 0.4 to 123; SD, 27.82). Table 1 describes the baseline characteristics of the study population.

### Revision Surgery

Figure 1 describes the various interventional paths after surgery. In 60% of all IBRs (*n* = 1196), revision surgery was necessary. Complications were the cause of revision in 54% (*n* = 646), elective reinterventions in 44% (*n* = 521), and oncologic reinterventions in 2% (*n* = 29). The mean number of revisions of all IBRs was 1.2 (range, 0 to 8; SD, 1.37). Due to the necessity of more than 1 revision surgery in some cases, a total of 2356 revisions were performed because of complications (43%; *n* = 1022), elective reinterventions (55%; *n* = 1288), or oncologic reinterventions (2%; *n* = 46). A final result of reconstruction was achieved in 0.0 to 114 months (median 5.2; SD, 23.31).

In breasts undergoing IBR using the DTI approach, 57% required a primary revision surgery, and in 30% of all DTI IBRs a second revision was necessary. Mean number of revisions of all DTI IBRs was 1.1 (range, 0 to 8; SD, 1.38). In the breasts using the 2-stage tissue expander method, the revision percentage was 99.3%, as implicitly, all breasts underwent at least 1 reintervention to replace their tissue expander for a permanent prosthesis, except for 1 patient who did not want her tissue expander to be replaced. A second revision, before or after replacement, was indicated in 52% of all 2-stage IBRs. Mean number of revisions of 2-stage IBRs was 1.7 (range, 0 to 8; SD, 1.05).

Univariable analysis showed a statistically significant association of revision surgery and patient-related risk factors, including age older than 44 years, BMI greater than 25 kg/m^2^, smoking, comorbidity, and specimen weight greater than 492 g (Table 2). Statistically significant treatment-related risk factors included mastectomy for therapeutic rather than prophylactic purpose, surgery performed by a nonstaff plastic surgeon rather than a staff plastic surgeon, implant volume greater than 422 g, and adjuvant radiotherapy. Nipple preservation, a DTI approach, and bilateral surgery demonstrated lower rates of revision surgery (Table 2). Multivariable analysis showed a higher odds for revision surgery in patients with age older than 44 years (OR, 1.23; 95% CI, 1.01 to 1.49), smoking (OR, 1.53; 95% CI, 1.09 to 2.15), specimen weight greater than 492 g (OR, 1.39; 95% CI, 1.03 to 1.86), implant volume greater than 422 g (OR, 1.95; 95% CI, 1.48 to 2.57), and adjuvant radiotherapy (OR, 1.51; 95% CI, 1.18 to 1.93). Nipple preservation (OR, 0.71; 95% CI, 0.58 to 0.88) and the DTI approach (OR, 0.01; 95% CI, 0.001 to 0.04) were associated with lower rates of revision surgery (Table 2).

**Table 2.** Univariable and Multivariable Analysis of Patient-Related and Treatment-Related Potential Risk Factors for Revision Surgery^a^

Characteristics	Univariable Analysis			Multivariable Analysis		
	OR	95% CI	*P*	OR	95% CI	*P*
Age >43.7 yrs	1.31	1.09–1.57	0.004^b^^,^^c^	1.23	1.01–1.49	0.042^b^
BMI >25.0 kg/m^2^	1.56	1.27–1.91	<0.001^b^^,^^c^	0.90	0.70–1.16	0.422
Tobacco use	1.62	1.18–2.23	0.003^b^^,^^c^	1.53	1.09–2.15	0.015^b^
Comorbidity^d^	1.33	1.05–1.69	0.016^b^	––	––	—
Specimen weight >491.5 g	2.39	1.97–2.90	<0.001^b^^,^^c^	1.39	1.03–1.86	0.029^b^
Therapeutic mastectomy	1.31	1.09–1.58	0.004^b^	—	—	—
Carcinoma ≥stage IIIA (invasive disease only)	0.85	0.62–1.17	0.328	—	—	—
Previous breast surgery^e^	1.24	0.95–1.61	0.109	—	—	—
Previous breast cancer surgery^f^	1.00	0.73–1.37	0.997	—	—	—
Neoadjuvant systemic therapy	1.07	0.88–1.29	0.508	—	—	—
Mantle field radiation	1.02	0.51–2.06	0.955	—	—	—
Bilateral mastectomy	0.83	0.69–0.99	0.038^b^	—	—	—
Nipple-areola complex preservation	0.60	0.50–0.72	<0.001^b^^,^^c^	0.71	0.58–0.88	0.001^b^
Direct-to-implant	0.01	0.001–0.07	<0.001^b^^,^^c^	0.01	0.001–0.04	<0.001^b^
Implant volume >421.5 g	1.91	1.59–2.29	<0.001^b^^,^^c^	1.95	1.48–2.57	<0.001^b^
Nonstaff oncologic surgeon	1.20	0.94–1.54	0.139	—	—	—
Nonstaff plastic surgeon	1.40	1.07–1.82	0.014^b^	—	—	—
Procedure time >142.9 min	1.17	0.98–1.40	0.091	—	—	—
Adjuvant systemic therapy	1.15	0.96–1.38	0.139	—	—	—
Adjuvant radiotherapy	1.46	1.15–1.84	0.002^b^^,^^c^	1.51	1.18–1.93	0.001^b^

a Odds ratios and confidence intervals of univariable and multivariable analysis of patient-related and treatment-related potential risk factors for revision surgery in 1196 breasts (60.1%).

b Statistically significant.

c Risk factors subjected to multivariable analysis using the Holm-Bonferroni correction method.

d General health factors, such as diabetes; cardiovascular, pulmonary, thyroid, or hematologic disorders; concurrent oncologic disease; or active inflammatory disease.

e Breast surgery in general, such as augmentation or reduction, including breast-conserving surgery.

f Breast-conserving surgery, such as wide local excision.

### Reconstruction Failure

In a total of 93.3% of the breasts (*n* = 1855), a breast was present at the end of follow-up (Fig. 1). In 221 breasts (11.1%), the reconstruction was lost during follow-up. This loss was temporary for 87 breasts (4.4% [87 of 1989]), as a secondary reconstruction was performed to restore the breast. An autologous or hybrid reconstruction was done in 67 breasts and an implant alone was inserted in 20 breasts. Of the remaining 134 losses (6.7% [134 of 1989]), patients refrained from pursuing further attempts at reconstruction (Fig. 1) because of complications (60% [*n* = 81]), patient preference (37% [*n* = 50]), or unknown or other reasons (2% [*n* = 3]).

Univariable analysis showed age older than 44 years, BMI greater than 25, comorbidity, specimen weight greater than 492 g, implant volume greater than 422 g, therapeutic mastectomy, surgery by a nonstaff oncologic surgeon compared with a staff oncologic surgeon, and both adjuvant systemic and radiation treatment to be significant risk factors for reconstruction failure. Nipple preservation and bilateral mastectomy were associated with lower rates of failure (Table 3). In multivariable analysis, only age older than 44 years (OR, 2.61; 95% CI, 1.67 to 4.06) and adjuvant radiotherapy (OR, 1.71; 95% CI, 1.12 to 2.61) remained significant predictors for reconstruction failure, whereas nipple preservation was associated with lower reconstruction failure rates (OR, 0.42; 95% CI, 0.28 to 0.64) (Table 3).

**Table 3.** Univariable and Multivariable Analysis of Patient-Related and Treatment-Related Potential Risk Factors for Reconstruction Failure^a^

Characteristics	Univariable Analysis			Multivariable Analysis		
	OR	95% CI	*P*	OR	95% CI	*P*
Age >43.7 yrs	3.45	2.29–5.21	<0.001^b^^,^^c^	2.61	1.67–4.06	<0.001^b^
BMI >25.0 kg/m^2^	1.63	1.14–2.35	0.008^b^	—	—	—
Tobacco use	1.56	0.94–2.59	0.086	—	—	—
Comorbidity^d^	1.78	1.20–2.64	0.004^b^	—	—	—
Specimen weight >491.5 g	1.90	1.34–2.71	<0.001^b^^,^^c^	1.30	0.89–1.90	0.171
Therapeutic mastectomy	2.20	1.45–3.34	<0.001^b^^,^^c^	1.04	0.61–1.77	0.881
Carcinoma ≥stage IIIA (invasive disease only)	1.27	0.75–2.17	0.375	—	—	—
Previous breast surgery^e^	1.27	0.79–2.02	0.323	—	—	—
Previous breast cancer surgery^f^	1.24	0.70–2.22	0.457	—	—	—
Neoadjuvant systemic therapy	1.27	0.89–1.82	0.187	—	—	—
Mantle field radiation	0.89	0.21–3.77	0.876	—	—	—
Bilateral mastectomy	0.49	0.34–0.71	<0.001^b^^,^^c^	0.88	0.56–1.38	0.581
Nipple-areola complex preservation	0.29	0.20–0.44	<0.001^b^^,^^c^	0.42	0.28–0.64	<0.001^b^
Direct-to-implant	2.69	0.99–8.10	0.056	—	—	—
Implant volume >421.5 g	1.66	1.17–2.37	0.005^b^	—	—	—
Nonstaff oncologic surgeon	1.59	1.05–2.42	0.029^b^	—	—	—
Nonstaff plastic surgeon	1.50	0.96–2.34	0.076	—	—	—
Procedure time >142.9 min	0.72	0.50–1.03	0.071	—	—	—
Adjuvant systemic therapy	1.65	1.16–2.34	0.005^b^	—	—	—
Adjuvant radiotherapy	2.10	1.43–3.07	<0.001^b^^,^^c^	1.71	1.12–2.61	0.013^b^

a Odds ratios and confidence intervals of univariable and multivariable analysis of patient-related and treatment-related potential risk factors for reconstruction failure in 134 breasts (6.7%).

b Statistically significant.

c Risk factors subjected to multivariable analysis using the Holm-Bonferroni correction method.

d General health factors, such as diabetes; cardiovascular, pulmonary, thyroid, or hematologic disorders; concurrent oncologic disease; or active inflammatory disease.

e Breast surgery in general, such as augmentation or reduction, including breast-conserving surgery.

f Breast-conserving surgery, such as wide local excision.

### Oncologic Follow-Up

A mastectomy was performed for invasive carcinoma in 947 breasts. These were divided into early disease (*n* = 739 [78%]) and (locally) advanced disease (*n* = 208 [22%]). A detailed overview of the molecular profile and Bloom-Richardson grading is provided in Supplemental Digital Content 2. (**See Table, Supplemental Digital Content 2**, which shows an overview of the tumor characteristics observed in a subgroup of 947 breasts that underwent nipple- or skin-sparing mastectomy and immediate implant-based breast reconstruction for invasive carcinoma. This includes, for example, the histopathologic tumor grade [Bloom-Richardson], hormone receptor status [estrogen receptor {ER}, progesterone receptor {PR}], and human epidermal growth factor receptor 2 [HER2] status, http://links.lww.com/PRS/H418.)

A total of 1427 of the 1512 women (94%) are alive; 31 (2.2%) have experienced a local recurrence and 42 (2.9%) have metastasized disease. Two women (3 IBRs) had metastatic breast cancer at the time of diagnosis. During follow-up, 85 women (5.6%) died between 5.9 and 96.1 months after mastectomy (mean, 43.5 months; SD, 22.08). Seventy-six women (89%) died because of metastasized breast cancer; the remaining 9 women (11%) died of other causes. In total, 118 women (7.8%) had metastasized disease at some point during follow-up.

## DISCUSSION

This long-term large patient cohort study showed that IBR is safe to perform, resulting in a high percentage of 93% having a breast at end of follow-up, which was achieved in only 1 procedure in 40% of all breasts. However, in 60%, revision surgery was necessary to achieve a final result, and 6.7% eventually ended up with permanent absence of a breast. Another 4.4% experienced temporary reconstruction loss (*n* = 87), and it is recognized that these losses have considerable psychologic impact. This 11.1% overall temporary or permanent reconstruction loss is within the range of previously reported 4% to 21% loss in other studies in the current literature.^10,18,21,30–32^ This study, however, focuses on permanent reconstruction failure. Age older than 44 years and radiotherapy are independent risk factors for both revision surgery and reconstruction failure. A history of smoking and large breast or implant size are risk factors for revision surgery, but not for reconstruction failure. Performing a nipple-sparing mastectomy, rather than a skin-sparing mastectomy, demonstrated lower rates of both outcomes. Moreover, in DTI IBRs, the desired result was achieved in a single procedure in 43%.

A wide range of revision surgery rates, varying from 11% to 85%, has been reported in previous studies^19,20,33^; our 60% revision rate is within this range even though our follow-up was extended. In addition, our revision surgery rate being toward the higher end may be attributable to our proactive approach in addressing suspected complications at an early stage to prevent potential failure. Likewise, our reconstruction failure rate of 6.7% is within the range of previously reported failure rates between 4.4% and 7.1%.^11,34^

Age older than 44 years is significantly associated with a 2.6-fold increased chance of reconstruction failure and 1.3-fold increased risk of revision surgery. Radiotherapy accounts for a 1.7- and 1.5-fold increased risk, respectively. Both factors are suggested by several studies.^18,20,21,35^ Changes in skin elasticity and tissue quality in older women, potentially affecting the long-term success of IBR, likely account for the increased rate of revision surgery to achieve a final result. In addition, with older age, priorities may shift, and may be associated with a different perspective on the necessity of having a breast, thus accepting a flattened chest in case failure occurs or choose to have the implant removed because of patient preference. This is supported by our finding that 66% of the older patient subgroup (103 of 157) did not undergo secondary reconstruction after reconstruction loss, whereas for the younger patient group, this value was 48% (31 of 64). Moreover, in 45% of the older women with reconstruction failure (46 of 103), the absence of a breast was attributed to patient preference. As for radiotherapy, it is well known that radiotherapy enhances capsule formation, causing more discomfort with the aesthetic result of the reconstruction, leading to requirement of several “touch-ups.”^6,18,36,37^ Adjuvant radiotherapy should not be considered a valid argument to advise against IBR; however, patients should be well informed preoperatively about the increased risk of revisions if radiotherapy is needed, as also stated by the National Institute for Health and Care.^3,38^

As described by others, smoking, specimen weight, and implant volume are predictors for revision surgery,^18,20,35^ but not for reconstructive failure.^21^ These risk factors are correlated with impaired wound-healing and hampering the perfusion of the skin, increasing the susceptibility to complications and need for revisions.^14,15^

In recent literature, there are conflicting findings regarding the safety of nipple-sparing mastectomy and IBR in terms of complications and reoperations. While concerns exist regarding an increased risk of nipple necrosis potentially leading to implant loss,^27,39,40^ several studies suggest otherwise, indicating a trend toward reduced rates of reoperations or implant loss.^18–20^ In our study, we further demonstrate that nipple preservation significantly decreases the risk of revision surgery and reconstruction failure. This correlation might be due to the fact that preservation of the nipple-areola complex results in a relatively larger skin envelope, allowing less tension during closure. Also, the breast contour is retained in its recognizable shape including the nipple-areola complex, therefore women might be more satisfied and less in need for “touch-ups.”^41^ Because nipple preservation has been shown to significantly improve the patient’s postoperative satisfaction and quality of life,^42,43^ we would recommend preserving the nipple if oncologically and technically feasible.

A DTI approach was performed in the majority of IBRs to rule out a second procedure and to potentially achieve a final result at once, which holds true in 43% of such IBRs. One may argue that a 2-stage approach rules out further secondary procedures aside from the conversion from tissue expander to prosthesis due to the possibility of integrating touch-ups or fat grafting during the implant exchange,^11^ but in our research, this group still required second reinterventions in 52% of the breasts compared to 30% of the patients receiving DTI. Others affirmed the DTI approach to be surgically safe, with less necrosis, infection, and implant explantation, and outperforming the 2-stage procedure.^10,11^ Moreover, nipple malposition is associated with skin elasticity, potential contracture of the skin, and the expansion process itself to change the volume of the tissue expander in a staged approach. This malpositioning can be prevented using the DTI method.^44^ Moreover, a DTI reconstruction does not increase the risk of reconstruction failure. It therefore seems justified to opt for a DTI approach when possible to reduce the extent of surgical procedures for a patient.

### Oncologic Follow-Up

In our study, a mastectomy for invasive breast cancer and IBR was performed in 78% for early breast cancer and 22% for (locally) advanced disease, which corresponds to the national figures (approximately 80% and 20%, respectively).^2^ In addition, our percentages of metastatic disease (7.8%) and local or regional recurrence (2.2%) are consistent with the national figures (the national metastatic disease rate is 14%; national figures for loco-regional recurrence rate range from 0 to 4%) for all women with breast cancer who underwent mastectomy.^2,45^ This confirms that IBR does not increase the risk of developing a metastasis or recurrence, and is therefore oncologically safe.

### Potential Limitations

A strength of this study is the extended follow-up of a large homogeneous cohort of nearly 2000 patients. Our approach ensures consistency and uniformity in surgical methodology, thereby enhancing the reliability and comparability of our outcomes. Although many variables were analyzed as potential factors contributing to both outcomes, there might be other confounding factors not included in this data set. Selection bias may have occurred, as we do not offer IBR to women who smoke, have a BMI greater than 25, and have large breasts, due to the increased risk of complications found in a previous study.^14^ Whereas others have reported that BMI is associated with an increased risk of revisions,^18–20,34^ this study was not able to confirm these results, potentially because the mean BMI of our cohort was relatively lower. Moreover, some continuous variables were dichotomized, as they had a normal distribution, to reduce group sizes. Statistical comparisons between age groups are not possible in our study due to insufficient sample sizes in certain groups (eg, age older than 65 years [*n* = 84]), thereby limiting our ability to draw meaningful conclusions regarding differences among them. It may be interesting to investigate a certain cutoff value for all continuous variables. Moreover, the mean age of our study population is lower than the average age at breast cancer diagnosis, as we only included patients undergoing IBR and not sole mastectomy, and because women treated in our comprehensive cancer center relatively often receive a prophylactic mastectomy due to genetic mutations. Outcomes were analyzed per breast, because failure or revision surgery is not related to the patient in case of bilateral surgery; however, we understand the impact of going through these procedures for the patient herself. Moreover, we recognize the lack of a standardized definition of revision surgery categorization across studies in the broader literature. Some reports only reported complications and implant loss as revision procedures^10,20^; others also scored touch-up procedures.^19^ We therefore categorized all extra surgeries women had to undergo under general anesthesia to prevent confusion regarding the definition of revision surgery. Future research should focus on establishing a standardized framework for classifying revision surgery.

## CONCLUSIONS

This comprehensive study evaluated the long-term outcome of IBR following mastectomy, and showed that in 93%, a breast was present. In 40%, no revision surgery was required. Older patients or patients with an indication for radiotherapy should be well informed about an increased risk of both revision surgery and reconstruction failure. Moreover, smoking, larger specimen weight, and greater implant volume are associated with an increased rate of revision surgery, but these factors are not responsible for more reconstruction failure. Nipple preservation is considered safe and even significantly reduces the risk of revision surgery and reconstruction failure. Using the DTI approach largely contributes to a revision percentage reduction and is the preferred method for IBR in our institute because it often prevents a patient from a definite secondary procedure and does not lead to increased rates of reconstruction failure. These factors play a crucial role in shared decision-making, guiding outpatient clinic consultations on IBR. Nevertheless, it is essential to acknowledge that these factors inform rather than discourage or exclude women from undergoing IBR.

## DISCLOSURE

The authors have no conflicts of interest to declare.

## Supplementary Material


