# Supplementary material for: Long-Term Outcomes of 1989 Immediate Implant-Based Breast Reconstructions: An Analysis of Risk Factors for Failure and Revision Surgery
Source: Plast Reconstr Surg. 2024 Sep 24;155(3):469–78. doi: 10.1097/PRS.0000000000011744 (PMC11845074; doi:10.1097/PRS.0000000000011744)
Supplement: Supplementary file 1 [file prs-155-469e-s001.pdf]

Table, Supplemental Digital Content 1. Prevalence and percentages of revision surgery (n=1,196) and reconstruction failure (n=134) in 1,989 breasts undergoing IBR after mastectomy, stratified by the presence or absence of potential risk factors.

		Revision surgery		***Reconstruction failure***	
**Characteristic**	**N**	**n**	**% (n/N)**	**n**	**% (n/N)**
Age (in years)					
> 43.7	1013	641	63%	103	10.2%
< 43.7	976	555	57%	31	3.2%
BMI (in kg/m^2)					
> 25.0	558	377	68%	51	9.1%
< 25.0	1431	819	57%	83	5.8%
Tobacco use					
YES	197	138	70%	19	9.6%
NO	1792	1058	59%	115	6.4%
Comorbidity[a]					
YES	375	246	66%	38	10.1%
NO	1614	950	59%	96	5.9%
Specimen weight (in grams)					
> 491.5	804	580	72%	74	9.2%
< 491.5	1185	616	52%	60	5.1%
Indication of mastectomy					
Therapeutic	1238	775	63%	104	8.4%
Prophylactic	751	421	56%	30	4.0%
Tumor category*					
≥ stage IIIA (advanced)	208	126	61%	20	9.6%
< stage IIIA (early)	739	475	64%	57	7.7%
Previous Breast Surgery[b]					
YES	284	183	64%	23	8.1%
NO	1705	1013	59%	111	6.5%
Previous Breast Conserving Surgery[c]					
YES	173	104	60%	14	8.1%
NO	1816	1092	60%	120	6.6%
Neo-adjuvant systemic therapy					
YES	697	426	61%	54	7.7%
NO	1292	770	60%	80	6.2%
Mantle field radiation					
YES	33	20	61%	2	6.1%
NO	1956	1176	60%	132	6.7%
Mastectomy laterality					
Bilateral	954	551	58%	43	4.5%
Unilateral	1035	645	62%	91	8.8%
Nipple-areolar complex					

Preservation	1048	570	54%	35	3.3%
Resection	941	626	67%	99	10.5%
Implant					
Direct-to-implant	1843	1051	57%	130	7.1%
Tissue-expander	146	145	99%[d]	4	2.7%
Implant volume (in grams)					
> 421.5	924	631	68%	78	8.4%
< 421.5	1065	565	53%	56	5.3%
Oncologic surgeon					
Non-staff	326	208	64%	31	9.5%
Staff	1663	988	59%	103	6.2%
Plastic surgeon					
Non-staff	283	189	67%	26	9.2%
Staff	1706	1007	59%	108	6.3%
Procedure time (in minutes)					
> 142.9	861	536	62%	48	5.6%
< 142.9	1128	660	59%	86	7.6%
Adjuvant therapy					
Systemic therapy					
YES	825	512	62%	71	8.6%
NO	1164	684	59%	63	5.4%
Radiotherapy					
YES	384	258	67%	43	11.2%
NO	1605	938	58%	91	5.7%

[a] = General health factors such as diabetes mellitus, cardiovascular, pulmonary, thyroid or hematologic disorders, concurrent oncologic disease, and active inflammatory diseases; [b] = breast surgery in general such as mamma-augmentation or –reduction and this also includes breast conserving surgery; [c] = such as wide-local excision or lumpectomy; [d] = one woman did not have her tissue-expander exchanged yet. * invasive disease only.
