# Supplementary material for: Long-Term Outcomes of 1989 Immediate Implant-Based Breast Reconstructions: An Analysis of Risk Factors for Failure and Revision Surgery
Source: Plast Reconstr Surg. 2024 Sep 24;155(3):469–78. doi: 10.1097/PRS.0000000000011744 (PMC11845074; doi:10.1097/PRS.0000000000011744)
Supplement: Supplementary file 2 [file prs-155-469e-s002.pdf]

Table, Supplemental Digital Content 3. Number and percentage of oncological characteristics in breasts that underwent the combined procedure for invasive mamma carcinoma (n=947).

Oncological characteristics	n	% (n/N)
**Early stage breast cancer, molecular profile**	**739**	**78**
- Triple positive	70	7.4
- ER+, PR+/-, Her2Neu –	485	51.2
- ER-, PR-, Her2Neu+	57	6.0
- Triple negative	127	13.4
**(Locally) advanced stage breast cancer, molecular profile**	**208**	**22**
- Triple positive	29	3.1
- ER+, PR+/-, Her2Neu –	125	13.2
- ER-, PR-, Her2Neu+	22	2.3
- Triple negative	32	3.4
**Bloom-Richardson grading**		
- Grade I	141	15
- Grade II	464	49
- Grade III	281	30
- Unknown	61	6.4
